# Sarcopenic obesity and physical performance in middle aged women: a cross-sectional study in Northeast Brazil

**DOI:** 10.1186/s12889-015-2667-4

**Published:** 2016-01-16

**Authors:** Mayle A. Moreira, Maria Victoria Zunzunegui, Afshin Vafaei, Saionara M. A. da Câmara, Tamyres S. Oliveira, Álvaro C. C. Maciel

**Affiliations:** 1 Department of Physiotherapy, Universidade Federal do Rio Grande do Norte, Avenida Senador Salgado Filho, S/N Caixa Postal 1524-Campus Universitário-Lagoa Nova CEP, Natal, RN 59072-970 Brazil; 2 Institut de Recherché en Santé Publique, School of Public Health, Université de Montréal, Québec, Canada; 3 Department of Public Health Sciences, Carruthers Hall, Queen’s University, Kingston, Canada

**Keywords:** Sarcopenic obesity, Sarcopenia, Obesity, Physical performance, Women, Middle-aged, Brazil

## Abstract

**Background:**

Sarcopenia and obesity have been independently associated with physical function decline, however little information is currently available on the relationship between sarcopenic obesity and physical performance, mainly in middle aged women. The present study aims to estimate the prevalence of sarcopenic obesity and to explore the relationship between sarcopenic obesity and physical performance in middle-aged women from Northeast Brazil.

**Methods:**

A cross-sectional study of women (40–65 years) living in Parnamirim, a city in Northeast Brazil (*n* = 491). Physical performance was assessed by grip strength, knee extensor and flexor strength (isometric dynamometry), gait speed, and chair stands. Using bioelectrical impedance analysis (BIA), appendicular skeletal muscle mass divided by height squared (kg / m^2^) was used to define sarcopenia. Waist circumference ≥ 88 cm was defined as abdominal obesity. Sarcopenic obesity was defined as the coexistence of obesity and sarcopenia. The physical performance outcomes were regressed in four groups defined by combinations of sarcopenia and obesity, adjusting for potential confounders (age, education and menopausal status).

**Results:**

Prevalence rates of the four obesity-sarcopenia groups were: Sarcopenic obesity (7.1 %), obesity (67.4 %), sarcopenia (12.4 %) and normal (13 %). Women with sarcopenic obesity had significantly lower grip strength, weaker knee extension and flexion and longer time to raise from a chair compared with non-obese and non-sarcopenic women (*p*.values < 0.001). Except for the chair stands, these statistically significant differences were also found between sarcopenic obese and obese women. There was no significant difference for gait speed across the four groups (*p* = 0.50).

**Conclusion:**

Sarcopenic obesity was present in 7 % of this population of middle-aged women from Northeast Brazil and it was associated with poor physical performance. Sarcopenic obesity may occur in middle-aged women with performance limitations beyond pure sarcopenia-related muscle mass or obesity alone.

## Background

Aging is a continuous process that involves physiological changes in multiple body systems resulting in reduced functional capacity [1]. The musculoskeletal system, involving bodily functions such as muscle contraction, movement and locomotion, is affected by loss of lean mass, particularly muscle mass (sarcopenia) [1].

Progressive muscle mass loss begins around 40 years and it is estimated that it decreases by 8 % every decade [2, 3]. After age 70, this loss rate increases to 15 % per decade [2, 3]. Although these changes have been found in the aging process of both men and women, a decrease in muscle mass does not occur at the same rate and age for both sexes. Study has reported a rapid strength loss in women around the age of 50, which is not observed in men until at least the age of 60 [4]. Menopause occurs during the same middle-aged period and it is associated with the natural decline of estrogen directly reflected in increasing visceral fat mass and decreasing bone density, muscle mass and strength [4]. A higher prevalence of obesity and a decrease in muscle mass are observed during menopause, and these health issues are separately related to a decline in function [5–7].

Furthermore, a higher rate of functional decline has been reported in subjects with sarcopenic obesity (SO), the co-existence of obesity and sarcopenia [8–10]. Lipid infiltration in muscle tissues seems to exacerbate sarcopenia, since accumulation of lipids both prevents incorporation of amino acids and reduces protein synthesis in the muscle [11]. For both men and women, having obesity and sarcopenia together due to a possible synergic effect can further decrease physical functioning [10, 12]. However, changes in body composition, such as obesity and sarcopenia, have been underdiagnosed; and when diagnosed they tend to be more frequent in older women than men [13].

Although sarcopenia and obesity have been independently associated with physical function decline [7, 14], little information is currently available on the relationship between SO and physical performance [10, 12]. Questions remain whether the effects of obesity on physical performance can be detected beyond those of sarcopenia among women that are both obese and sarcopenic. Furthermore, to our best knowledge, no study has examined this relation using objective and valid measures of physical performance and body composition in middle-aged women.

The number of obese people is increasing in Brazil and the current prevalence of excess weight is at least 3-times higher than that of undernutrition [15]. To illustrate, in two recent studies conducted in the south of Brazil [16, 17], two thirds of middle-aged women had abdominal obesity; however, these studies did not look at sarcopenia.

The present study aims to estimate the prevalence of sarcopenic obesity and to explore the relationship between sarcopenic obesity and physical performance in middle-aged women from Northeast Brazil.

## Methods

### Study population/data collection

This cross-sectional study took place in Parnamirim, a city in the Northeast of Brazil located in Natal’s metropolitan region, the capital of Rio Grande do Norte (RN) state. This city has around 200,000 inhabitants, distributed across 123.5 km^2^, and is 100 % urbanized.

In this paper, we used baseline data from an ongoing longitudinal research [18]. The longitudinal study aims to analyze the influence of hormone levels on sarcopenia (muscle loss) and physical functioning. The present data were collected between April and November of 2013.

The study population was composed of women living in Parnamirim (40–65 years). Women were recruited by advertisements in all primary care centers across the city. Brazil has universal health care coverage, and all citizens are entitled to register at local neighborhood health centers. The coverage of the primary health care is system is estimated to be 74.5 % in Parnamirim [19]. A convenience sample of five hundred women comprised the baseline study sample. To be eligible to participate in the study, women had to have at least one ovary, with no pituitary or thyroid alterations, without serious neurological diseases that could prevent them from responding to questions, and be free of painful conditions that could compromise the measurement of physical performance outcomes. Women who refused to complete all data collection stages were excluded. Our final sample consisted of 491 women.

All women were evaluated in a single community health center of Parnamirim, RN. The assessments were conducted by trained interviewers using standard protocols.

### Ethics

All participants were informed of the objectives and procedures of the research at first contact and signed an informed consent form. The study protocol received approval from the Ethics and Research Committee of the Federal University of Rio Grande do Norte (number: 387.737).

### Procedures

#### Physical performance

Physical performance was assessed by five tests: grip strength, knee extension and flexion strength, gait speed and chair stands. These constituted the dependent variables for this study. Grip strength: the dominant hand was evaluated with a Jamar® dynamometer in the second handle position [20]. The participant was positioned, as recommended by the American Society of Hand Therapists [21], seated with shoulder fully adducted and neutrally rotated, elbow flexed at 90° and forearm in a neutral position. The participants were requested to squeeze the dynamometer with maximal isometric effort without any other body movement, for five seconds. The test was performed three times, with a one-minute interval between measurements. The mean of these three trials was used for analysis.

Knee flexion and extension strength (dominant limb) were measured with the Hoggan® portable dynamometer, MicroFET2® model (isometric dynamometer). The evaluation of extensors was performed with the subject positioned in the prone position on a stretcher, with the knee flexed at 90° and the thigh fixed to the stretcher by an inelastic band. The dynamometer was fixed on the anterior surface of the lower limb, on the line immediately proximal to the malleolus. The evaluation of flexors was carried out in the same position, however with the extended knee and the dynamometer positioned on the posterior surface of the lower limb, immediately above the line of the malleolus. Three maximal isometric contractions of five seconds were requested with one-minute intervals between measurements. The arithmetic mean of the three measurements in kilograms/force (kgf) was considered for analysis [22].

For gait speed, a 4-m walk at the subject’s usual pace was timed. The faster of two walks was used. Gait speed was calculated in meters per second [23, 24].

For the chair stand test, participants were asked to stand up and sit down five times as quickly as possible with arms folded across their chests and were timed in seconds from the initial sitting position to the fifth standing position [23, 24].

#### Classification of obesity, sarcopenia and sarcopenic obesity

Women with a waist circumference ≥ 88 cm were considered obese, as proposed by Brazilian obesity guidelines [25]. Janssen et al. [26] have described their results suggesting that waist circumference is a better marker of health risk than BMI, and consequently a greater emphasis should be placed on waist circumference in the obesity classification system. Waist circumference may provide an estimate of increased abdominal fat even in the absence of a change in BMI, avoiding some misclassification [26]. Waist circumference (cm) was measured using a “fiber glass” metric tape (divisions of 1 mm) and the measurement point was the mid-way point between the lowest rib and the iliac crests while the subject was standing at the end of normal expiration [27]. Participants were positioned with their feet together, arms crossed over their chest and instructed to relax.

All participants underwent body composition analysis using the same InBody R20 bioelectrical impedance analysis (BIA) machine in compliance with the manufacturer’s instructions. This BIA model is composed of eight electrodes, two for each hand and two for each foot, and performs analysis in 20 and 100 KHz with a current of 250 μA. The body composition was automatically calculated according to predictive equations provided by the manufacturer. The skeletal muscle mass index (SMI) was assessed considering SMI = ASM (sum of appendicular skeletal muscle mass) (kg) / height^2^ (m). BIA presents good correlation with predictions made by magnetic resonance imaging [28] and Dual-energy X-ray absorptiometry-DEXA [29]. Although it is not the gold standard, it is used in research [30–32], it is a reliable alternative for evaluation of body composition, and it has advantages of being portable, lower cost and not exposing subjects to radiation.

Using the skeletal muscle mass index (SMI) measured by bioelectrical impedance, women were classified as sarcopenic if they had SMI values below the 20th percentile of the sample studied (<6.08 kg / m^2^) [33, 34]. SO was defined as the coexistence of obesity and sarcopenia [8, 9]. Next, the women were classified into four groups: Obese, sarcopenic, sarcopenic obese, and lastly, non-sarcopenic non-obese women, herein called normal women.

##### Covariates

Age, sex, marital status, education, income, physical activity and menopausal status were identified as potential confounders of the association between obesity, sarcopenia and physical function, according to the literature on risk factors for physical performance [18, 35, 36].

##### Demographic and socioeconomic variables

Age was evaluated in years. Education was assessed as years of schooling and then categorized into three groups: less than basic education (up to seven years), between basic and secondary (more than seven and less than eleven years), and secondary or more (eleven years and over). Marital status was classified according to the self-reported presence or absence of a common law union. The race/ethnic group was also declared by the participant as white, black or mixed race (pardo).

Family income was categorized according to the Brazilian minimum monthly wage (MW), which is the lowest remuneration that employers may legally pay workers. Theoretically, this MW should be enough to supply the normal needs of food, housing, clothing, hygiene and transport for a family. At the time of the interview, the MW was R$678.00 (Brazilian Real) per month (approximately $250US dollars/month). However, according to the Statistics and Socioeconomic Studies Department of Brazil (DIEESE) [37], this minimum salary is insufficient. In our study, family income was dichotomized as less than 3 MW and 3 MW or more (3 MW is R$2.034 Brazilian Real, which is equivalent to US$750 dollars). The choice of 3 MW was based on what is considered to be the poverty threshold in the Northeastern region of Brazil [18].

##### Physical activity

Women were asked if they regularly practiced physical activity. In the event of an affirmative answer, they were asked further about frequency, duration and type of physical activity. To register sedentary activity, women were asked to report how many hours they remained seated in a regular day. This variable was dichotomized into 4 h/day or less and more than 4 h/day [38]. For walking behavior, women were instructed to report how many days per week, and for how long per day they had walked for more than 10 min without stopping during the last week. A walking indicator was constructed to distinguish women walking 90 min/week or more [18].

##### Menopausal status

Menopausal status was defined using the classification STRAW (Stages of Reproductive Aging Workshop classification) [39] or a self-report of hysterectomy. Women were classified into five groups: premenopausal (regular menses), perimenopausal (irregular menses, with differences in cycle length over seven days or amenorrhea up to one year), postmenopausal (absence of menses for over one year), hysterectomy carried out before 40 years old or after age 40.

##### Body mass index, hip-waist ratio and skeletal muscle mass index

Weight (kg) was measured by the Wiso® W903 digital scale and height (m) by a stadiometer. To measure hip circumference (cm), a fiber glass® metric tape was used and the hip measurement was the largest horizontal circumference around the hips. Waist-hip ratio was calculated by dividing these variables (waist/hip) [27]. Body mass index (BMI) was calculated by dividing body weight by height squared (kg/m^2^). The skeletal muscle mass index (SMI) was assessed considering SMI = ASM (sum of appendicular skeletal muscle mass) (kg) / height^2^ (m) [33], by an InBody R20 BIA machine.

### Data analysis

We started the data analysis with a descriptive analysis of the sample. Means and standard deviations of quantitative variable frequencies of categorical variables were estimated. We utilized Analysis of Variance (ANOVA) methods to compare the mean of physical performance measures across the four normal, obese, sarcopenic and sarcopenic obese groups. We used the Kolmogorov-Smirnov test to evaluate the normality of the data. The post-hoc multiple comparisons Bonferroni test was used to verify which of these four groups were significantly different from each other. Finally, we constructed multivariate linear regression models to estimate the mean of physical performance measures in each of the four groups, adjusting for potential confounders. The potential confounders were identified according to the literature and bivariate analyses (*p* < 0.20) of their relationships with physical performance measures. All analyses were conducted using SPSS version 20.0 (SPSS, Chicago, IL, USA).

## Results

The total sample characteristics are shown in Table 1. The sample consisted of 491 women with a mean age of 49.95 ± 5.56 years. Approximately 70 % were married or living in common law unions and 55 % were of mixed ethnic groups. Nearly 41 % had less than primary education, and 70 % were living below the poverty threshold of 3 MW. Almost 75 % reported no regular physical activity.

**Table 1:** Characteristics of the total sample

Variables (*n* = 491)	*n*	% or mean ± SD
Age	491	49.95 ± 5.56
Education		
Primary education	205	41.8
Between primary and secondary	204	41.5
Secondary or more	82	16.7
Family income		
≥ 3 MW	146	29.7
< 3 MW	345	70.3
Common law marriage		
No	139	28.3
Yes	352	71.7
Race		
White	192	39.3
Black	27	5.5
Pardo (Mixed race)	270	55.2
Regular Physical Activity		
No	360	73.5
Yes	130	26.5
Walking (min/week)		
< 90	264	53.8
≥ 90	227	46.2
Sitting time per day		
4 h or less	321	65.5
More than 4 h	169	34.5
Menopausal status		
Premenopausal	102	21.7
Perimenopausal	152	32.4
Post-menopausal	130	27.7
Hysterectomy before 40 years	44	9.4
Hysterectomy after 40 years	41	8.7
Weight (Kg)	491	68.32 ± 12.46
Height (m)	491	1.54 ± 0.06
BMI (Kg/m^2^)	491	28.96 ± 4.83
Waist circumference (cm)	491	95.12 ± 10.73
SMI (kg/m^2^)	491	6.78 ± 0.87
Grip Strength (Kgf)	491	25.86 ± 5.35
Knee Flexion Strength (Kgf)	491	14.90 ± 4.86
Knee Extension Strength (Kgf)	491	16.51 ± 4.18
Gait speed (m/s)	491	0.99 ± 0.18
Chair Stand (s)	491	10.20 ± 2.04

*MW* minimum wages, *BMI* body mass index, *SMI* skeletal muscle mass index

Table 2 shows the sample distributions for categorical variables by groups of obese women (67.4 %), normal (13 %), sarcopenic (12.4 %) and sarcopenic obese (7.1 %). No significant differences between the four groups were found according to socioeconomic status or health behaviors. SO was more prevalent among the women who had had a hysterectomy and among the premenopausal women than among peri and post-menopausal women (*p* < 0.05).

**Table 2:** Sample characteristics among obese, normal, sarcopenic and sarcopenic obese groups

Variables	Obese		Normal		Sarcopenia		Sarcopenic obesity		*p* value
	*n* = 331 (67.4 %)		*n* = 64 (13.0 %)		*n* = 61 (12.4 %)		*n* = 35 (7.1 %)		
n (%)									
Age									
≤ 45 years	67	(67.6 %)	10	(10.1 %)	14	(14.1 %)	8	(8.1 %)	0.88
46–49 years	96	(65.8 %)	24	(16.4 %)	17	(11.6 %)	9	(6.2 %)	
50–54 years	109	(69.9 %)	19	(12.2 %)	19	(12.2 %)	9	(5.8 %)	
>55 years	59	(65.6 %)	11	(12.2 %)	11	(12.2 %)	9	(10.0 %)	
Education									
Less than primary education	148	(72.2 %)	26	(12.7 %)	18	(8.8 %)	13	(6.3 %)	0.09
Between primary and secondary	135	(66.2 %)	25	(12.3 %)	26	(12.7 %)	18	(8.8 %)	
Secondary or more	48	(58.5 %)	13	(15.9 %)	17	(20.7 %)	4	(4.9 %)	
Family income									
≥ 3 MW	97	(66.4 %)	23	(15.8 %)	19	(13.0 %)	7	(4.8 %)	0.41
< 3 MW	234	(67.8 %)	41	(11.9 %)	42	(12.2 %)	28	(8.1 %)	
Common law									
No	86	(61.9 %)	25	(18.0 %)	19	(13.7 %)	9	(6.5 %)	0.18
Yes	245	(69.6 %)	39	(11.1 %)	42	(11.9 %)	26	(7.4 %)	
Race									
White	129	(67.2 %)	25	(13.0 %)	24	(12.5 %)	14	(7.3 %)	0.26
Black	24	(88.9 %)	1	(3.7 %)	0	(0.0 %)	2	(7.4 %)	
Pardo (Mixed race)	177	(65.6 %)	37	(13.7 %)	37	(13.7 %)	19	(7.0 %)	
Physical activity									
No	241	(66.9 %)	47	(13.1 %)	45	(12.5 %)	27	(7.5 %)	0.96
Yes	89	(68.5 %)	17	(13.1 %)	16	(12.3 %)	8	(6.2 %)	
Walking (min/week)									
<90	179	(67.8 %)	28	(10.6 %)	38	(14.4 %)	19	(7.2 %)	0.22
≥ 90	152	(67.0 %)	36	(15.9 %)	23	(10.1 %)	16	(7.0 %)	
Sitting time per day									
4 h or less	216	(67.3 %)	36	(11.2 %)	45	(14.0 %)	24	(7.5 %)	0.22
More than 4 h	114	(67.5 %)	28	(16.6 %)	16	(9.5 %)	11	(6.5 %)	
Menopausal status									
Premenopausal	58	(56.9 %)	18	(17.6 %)	14	(13.7 %)	12	(11.8 %)	0.05
Perimenopausal	113	(74.3 %)	14	(9.2 %)	19	(12.5 %)	6	(3.9 %)	
Post-menopausal	91	(70.0 %)	15	(11.5 %)	18	(13.8 %)	6	(4.6 %)	
Hysterectomy before 40 years	26	(59.1 %)	9	(20.5 %)	3	(6.8 %)	6	(13.6 %)	
Hysterectomy after40 years	24	(58.5 %)	6	(14.6 %)	6	(14.6 %)	5	(12.2 %)	

In multiple comparisons using Bonferroni correction, all differences in body distribution variables across the four groups were significant, except differences in BMI between normal women (25.18 ± 2.06) and sarcopenic obese (26.23 ± 2.68) (*p* = 0.98); in waist/hip between obesity and SO (*p* = 1.00); and in skeletal muscle mass between sarcopenia and SO (*p* = 1.00) (Table 3).

**Table 3:** Quantitative variables of body composition according to the groups (obesity and sarcopenia)

Variables	Obese	Normal	Sarcopenia	Sarcopenic obesity	*p* value
	*n* = 331 (67.4 %)	*n* = 64 (13.0 %)	*n* = 61 (12.4 %)	*n* = 35 (7.1 %)	
	mean ± SD	mean ± SD	mean ± SD	mean ± SD	
SMI (Kg/m^2^)	7.16 ± 0.69	6.60 ± 0.44	5.54 ± 0.41	5.66 ± 0.30	<0.001
BMI (Kg/m^2^)	31.14 ± 4.05	25.18 ± 2.06	22.58 ± 2.06	26.23 ± 2.68	<0.001
Waist circumference (cm)	100.25 ± 8.16	83.13 ± 5.88	80.98 ± 5.10	93.04 ± 3.34	<0.001
Waist-hip ratio	0.92 ± 0.05	0.84 ± 0.07	0.88 ± 0.04	0.91 ± 0.06	<0.001

*SMI* skeletal muscle mass index, *BMI* body mass index

Table 4 summarizes the results of adjusted and unadjusted analyses of the physical performance variables across the groups. Age, education and menopausal status were considered potential confounders according to the literature and the bivariate analyses, since they were associated with obesity/sarcopenia groups (*p* < 0.20). There were significant differences between groups in strength (grip strength, knee flexor and extensor strength) and timed chair stands: women classified as sarcopenic obese had the worst physical performance, with lowest mean values for strength variables and the longest time for the timed chair stands. Average gait speed was not different across groups (*p* = 0.50).

**Table 4:** Mean levels (unadjusted and adjusted) of physical performance according to groups (obesity and sarcopenia)

Variables	Obese	Normal	Sarcopenia	Sarcopenic obesity	
	*n* = 331 (67.4 %)	*n* = 64 (13.0 %)	*n* = 61 (12.4 %)	*n* = 35 (7.1 %)	
	Mean (95 % CI)				*p* value
Grip Strength (Kgf)					
Unadjusted	26.49 (25.91–27.07)	26.91 (25.40–28.40)	23.54 (22.61–24.47)	22.07 (20.66–23.47)	<0.001^b^
Adjusted^a^	26.94 (26.26–27.62)	26.93 (25.64–28.22)	23.65 (22.33–24.97)	21.88 (20.18–23.58)	<0.001
Knee Flexion Strength (Kgf)					
Unadjusted	15.48 (14.94–16.02)	15.47 (14.33–16.60)	13.19 (12.16–14.22)	11.26 (9.97–12.55)	<0.001^b^
Adjusted^a^	15.73 (15.08–16.39)	15.59 (14.35–16.83)	13.14 (11.89–14.39)	11.41 (9.73–13.08)	<0.001
Knee Extension Strength (Kgf)					
Unadjusted	17.06 (16.60–17.51)	16.80 (15.68–17.91)	14.70 (13.87–15.52)	13.91 (12.64–15.18)	<0.001^b^
Adjusted^a^	17.57 (17.01–18.13)	16.98 (15.93–18.04)	14.81 (13.74–15.87)	14.16 (12.73–15.58)	<0.001
Gait speed (m/s)					
Unadjusted	0.99 (0.96–1.00)	1.00 (0.95–1.04)	0.99 (0.94–1.03)	0.94 (0.86–1.01)	0.50
Adjusted^a^	1.01 (0.99–1.03)	1.01 (0.96–1.06)	1.00 (0.96–1.05)	0.96 (0.90–1.02)	0.50
Chair Stand (s)					
Unadjusted	10.29 (10.06–10.51)	9.79 (9.33–10.24)	9.71 (9.21–10.20)	11.00 (10.18–11.82)	0.009^c^
Adjusted^a^	10.11 (9.83–10.38)	9.71 (9.18–10.24)	9.76 (9.22–10.29)	10.89 (10.18–11.60)	0.03

^a^Adjusted for age, education and menopausal status. In multiple comparisons with Bonferroni corrections (p < 0.05)

^b^Normal ≠ sarcopenia and sarcopenic obesity; sarcopenia ≠ obese; obese ≠ sarcopenic obesity

^c^Normal ≠ sarcopenic obesity; sarcopenia ≠ sarcopenic obesity

Multiple comparisons showed that in general, women with sarcopenia had significantly worse performance than obese women (grip strength *p* < 0.001; knee flexor strength *p* < 0.003; knee extensor strength *p* < 0.001). SO women showed lower mean values than obese women in all strength variables (*p* < 0.001) (Table 4). It also appears that obesity and sarcopenia impose a synergic ill effect on physical performance. It is best demonstrated when we look at differences in times for chair stands. For sarcopenic obese women, it took an average of 1.18 (that is, 10.89–9.71) seconds longer than normal women to stand, which is more than what would be expected by adding the 0.40 (that is, 10.11–9.71) excess time of obese women compared to normal women, and 0.05 (that is, 9.76–9.71), the corresponding excess time for sarcopenic women.

## Discussion

### Summary of results

Sarcopenic obesity (SO) was relatively frequent and associated with poor physical performance in middle-aged women from Northeast Brazil. Women with SO had significantly lower grip strength and knee extension and flexion strength when compared with normal and obese women. Although not statistically significant, women with SO tended to have lower values in the three muscle strength indicators (grip strength, knee extension and flexion strength), compared with sarcopenic non-obese women. Concerning chair stands, sarcopenic obese women did not differ from obese women, but it took them longer to raise from a chair than non-obese women with or without sarcopenia.

### Prevalence findings

The prevalence of SO in our study was 7.1 %. Due to the lack of a standard definition for SO, it is difficult to compare its prevalence across populations. In addition, in the eyes of clinicians and researchers, SO is more a problem for older women and has seldom been studied in middle-aged women. The few existing studies report wide-ranging results. A Korean study found that the prevalence of SO varies from 0.8 to 11.8 % in women between 40 and 59 years, according to different indices of definition [40]. Among older women, the prevalence of SO has been as high as 19.2 % in Taiwanese women (mean age: 63.3 years) [41] and 21.5 % in Brazilian women (mean age: 67.2 years) [42]. Furthermore, the prevalence rate of SO in Canadian women aged 68–82 years old was 10.8 % [43], close to the prevalence of 9.2 % in post-menopausal European women (mean age 57.4 years) [44]. The muscle mass can be influenced by age, height, body weight and ethnicity, but also by nutrition and life course exposure to social and economic adversity [45, 46]. Ethnicity and social and economic life course adversity are often difficult to separate. As an illustration, elderly Mexicans have less muscle and greater total fat than New York dwelling Caucasians, but researchers were unable to separate the ethnic and socioeconomic influences [47].

Obesity is increasing in Brazilian female adolescents and women without a marked socioeconomic gradient [15]. The high prevalence of obesity in our study population is in the range of what has been reported in the literature for adult women in Northeast Brazil [48–50]. Andrade et al. [51] found a similar prevalence of 55.5 % (≥88 cm), based on waist circumference in women with a mean age of 39.9 years [51].

Concerning sarcopenia, in a study conducted in France, the prevalence was 9 % for individuals between 45 and 54 years, 13.5 % in the group aged between 55 and 64 years, and reaching 64.3 % in those aged 85 years or older, with no difference between men and women, indicating that sarcopenia is already present in middle-aged populations. Sarcopenia was negatively associated with BMI, although the authors did not report on SO [52].

### Findings on grip strength

Low muscle mass predisposes physical function decline and increases the risk of falls, disability, poor quality of life and mortality [53, 54]. It has been suggested that SO could exert a synergistic impact on physical performance of the elderly [12].

In concordance with a recent Brazilian study of older women, we found that SO is associated with reduced grip strength [1]. Two additional studies support our results. First, Lim et al. [55] found significant difference between these four sarcopenic-obesity groups in older Asian women from Singapore and concluded that obesity coupled with sarcopenia results in worsening of performance and strength parameters, including grip strength [55]. Second, Baumgartner et al. [56] observed the lowest grip strength among sarcopenic obese men and women of 60 years and greater in a United States population, corroborating our results [56].

Previous research indicates that obese people have higher absolute muscle strength and a similar or higher ‘strength to muscle size ratio’ compared to their lean counterparts [57]. However, the disabling effect of excess fat mass may reduce motor performance of complex motor tasks that require body mass support or mobilization [57, 58]. Non-sarcopenic and sarcopenic obese women may be considered an apparently homogenous group of obese women; however, they have very different physical performance because of their muscle mass. We are in agreement with Newman et al. [54], who highlighted the importance of detecting obese individuals who do not appear to be sarcopenic, but in fact their muscle mass is reduced for their body size and this reduction is masked by obesity [54].

### Lower limb strength findings

We found lower limb strength differs between the groups, with obese women showing similar results as normal women, while sarcopenic obese women presented the worst performance. Miyatake et al. [59] showed the absolute isometric strength of knee extensors was higher in obese adults (20–60 years) compared to non-obese controls [59]. Moreover, Rolland et al. [60] showed that active obese elderly produced higher relative strength per unit muscle size than non-obese, while sedentary obese older adults had similar muscle strength compared to non-obese [60]. Consequently, favourable adaptations to excess body mass on muscle function might depend on the maintenance of sufficient physical activity during aging. However in our study, the four groups reported similar practice of regular physical activity, sedentary and walking behaviours. The reason that in our sample obese participants showed the same strength as normal women might be due to the fact that all have the same level of physical activity (Table 2).

### Findings on chair stands

Unlike strength variables, obese and sarcopenic obese women took longer time for chair stands compared with normal and sarcopenic women. Chair stands is an activity in which the body sequentially displaces itself in the shortest time possible and requires more than strength; it also involves muscle power and coordination [57, 58]. For chair stands, the SO women performed even worse than the sarcopenic, which corroborates reviewed studies stating that central fat and relative loss of fat-free mass can determine the health risk associated with obesity at older ages [61]. Sarcopenia and obesity may act synergistically, leading to functional and metabolic changes [62], and our results provide evidence for this synergism by showing the decline in the chair standing performance in SO participants. Waters et al. [63] found several functional deficits in the SO group relative to normal, sarcopenic and obese women, with the worst performance on the chair stands test by SO. However, Lim et al. [55] found no significant difference across groups in this test, despite a longer observed time in the SO group [55].

### Gait speed findings

We did not find any significant differences in gait speed across groups despite the fact that high intermuscular fat in the thigh is a known predictor of gait-speed decline [64]. Fat infiltration into muscle contributes to the loss of mobility associated with aging, and decreasing thigh muscle area is also predictive of decline in gait speed [64].

The observed lowest mean values of gait speed in women with SO, albeit non-significant, may reflect on their functional capacity and quality of life in the near future. While Meng et al. [10] found an absence of association between gait speed and the groups (obesity, sarcopenia and SO) in older women [10], Lim et al. [55] reported lower gait speed in sarcopenic obese older Asian men and women (≥65 years) from Singapore [55].

### Limitations and strengths

Our research has some limitations. The participants were middle-aged women from Northeast Brazil, therefore caution should be used in inferring the results to other populations. Firstly, cross cultural comparisons of prevalence and distribution of SO are difficult due to variability in body sizes and body composition, as well as the lack of standard protocols in diagnosis of SO. This diagnosis is usually based on combinations of body composition indices and muscle mass with varying population specific cut-off points, as is usually recommended to study of diverse populations [47]. Second, we used a convenience sample and our participants might have been healthier than the average target population. The possibility of healthy volunteer bias cannot be ruled out. However, education and income of the study sample is similar to the census data for the women of similar ages residing in the city of Parnamirim. Lastly, there is a possibility of information bias due to self-reported measures of health behaviors; however, since there is no reason to think that this bias is related to physical performance, the misclassifications are probably non-differentiatial.

The main strength of this study is its focus on a very rarely studied condition-sarcopenic obesity-in lower income middle-aged women. Objective measurement of physical performance and body composition by valid, non-invasive, and inexpensive tools is another strength of this study.

### Relevant clinical implications

Obesity associated with sarcopenia seems to worsen physical performance, beyond what has been reported by obesity or sarcopenia alone. Although the differences between the groups were relatively small, it has been shown in the literature that changes in chair stand performance, for example, provide a marker of current health and were predictive of mortality among middle-aged women in a ten years period [65], and also predictive of disability in older adults [23].

Investigation of sarcopenia and sarcopenic obesity should be considered in the clinical assessment of menopausal women. Furthermore, preventive measures and/or rehabilitation in relation to SO need to be implemented to decrease the ill effects of SO.

## Conclusion

Sarcopenic obesity was relatively frequent in middle-aged women from Northeast Brazil, and it was associated with poor physical performance. Sarcopenic obesity may occur in middle-aged women with subsequent performance limitations beyond sarcopenia—related muscle mass or obesity alone.

The number of obese women is rapidly increasing in many Latin America countries and a substantial proportion of middle-aged women may be affected both by obesity and low muscle mass. It is anticipated that SO will be a future public health burden.

Most studies on SO have been conducted in older adults. Further research in the etiology and onset of SO in middle-aged women is needed. These studies may include longitudinal data to confirm our cross-sectional findings. Furthermore, an assessment of the efficacy of interventions to maintain muscle mass and strength in this age group should be carried out.

## Abbreviations

BIA: bioelectrical impedance analysis
BMI: body mass index
MW: monthly wage
SMI: skeletal muscle mass index
SO: sarcopenic obesity
WHO: world health organization
STRAW: stages of reproductive aging workshop classification
